# Tripartite Motif Protein 6 Promotes Colorectal Cancer Cell Migration and Metastasis *via* SOCS2-STAT3 Signaling

**DOI:** 10.3389/fonc.2021.695525

**Published:** 2021-09-13

**Authors:** Hongjian Zhao, Junjun Huang, Ming Chen, Baoru Li, Xinran Chen, Mingqing Zhou

**Affiliations:** Department of General Surgery, Zhabei Central Hospital of Jing'an District, Shanghai, China

**Keywords:** colorectal cancer, metastasis, TRIM6, STAT3, SOCS2

## Abstract

Colorectal cancer (CRC) is one of the leading causes of cancer death worldwide, with most mortalities being caused by metastases. However, the underlying molecular mechanism of CRC metastases remains largely unknown. Emerging evidence has shown the role of the tripartite motif family, especially tripartite motif protein 6 (TRIM6), in carcinogenesis. In this study, we used CRC cell lines with TRIM6 knockdown and overexpression to investigate the function of TRIM6 in CRC metastasis. We found that TRIM6 promotes CRC cell migration and invasion both *in vitro* and *in vivo*. TRIM6 knockdown slows down the migration and invasion processes, whereas TRIM6 overexpression accelerates CRC cell migration and invasion. TRIM6 is potentially the upstream regulatory factor for signal transducer and activator of transcription 3 (STAT3) *via* the suppressor of cytokine signaling 2 (SOCS2). A total of 70 samples from patients with CRC further confirmed that TRIM6 expression level is positively correlated with STAT3 phosphorylation and negatively correlated with SOCS2 expression. Therefore, TRIM6 could be a potential therapeutic target for CRC metastasis.

## Introduction

Colorectal cancer (CRC) accounts for 9% of all cancers worldwide, being the second most common cancer in women and the third most common cancer in men (1, 2). Majority (90%) of the CRC cases are adenocarcinomas originating from the colonic and rectal epithelium (3), while CRC metastasis in the liver is the leading cause of CRC mortality (4). Similar to other cancers, CRC metastasis is a highly integrated process, involving processes such as cancer cell migration, adhesion, and invasion (5). These processes require precise molecular and cellular regulation, including cell adhesion molecules (6), cell–matrix interactions (7), and epithelial–mesenchymal transition (EMT), among others (8). Multiple signaling pathways, including the transforming growth factor beta (TGF-β) (9), Wnt (10), Notch (11), signal transducer and activator of transcription 3 (STAT3) (12), and PI3K/AKT (13), have been linked to cancer metastasis. However, the underlying mechanisms of CRC metastasis remain largely unknown.

The tripartite motif family, also known as the TRIM family, consists of 70 members, most of which have the E3 ubiquitin ligase activity (14). Accumulating studies have indicated its function in regulating carcinogenesis and metastasis (14–16). Tripartite motif protein 6 (TRIM6) belongs to the TRIM family with the E3 ubiquitin ligase activity (17). Besides its antiviral effect (17–19), TRIM6 has also been selectively expressed in embryonic stem (ES) cells. It attenuates c-Myc activity *via* a direct interaction. Downregulating TRIM6 in mouse ES cells results in the increased transcriptional activity of c-Myc and ES cell differentiation. Regarding its mechanism, TRIM6 functions as a ubiquitin ligase for c-Myc, allowing it to maintain c-Myc at an appropriate level (20). Recently published data indicate that TRIM6 promotes CRC cell proliferation (16). This finding indicates the potential function of TRIM6 in carcinogenesis and cancer metastasis.

In this study, we investigated the role of TRIM6 in CRC cell migration and invasion and, thus, found that TRIM6 promotes CRC cell migration and invasion. We identified that the STAT3 singling pathway was positively correlated with TRIM6 expression in CRC samples *via* a gene set enrichment analysis (GSEA). STAT3 is a transcription factor and is activated by phosphorylation (p-STAT3). STAT3 promotes proliferation, angiogenesis, migration, and invasion in its constitutively active form (21–24). Reports have shown that STAT3-negative regulators, such as SHP-1, SHP-2, SOCS1, SOOCS2, SOCS3, and PIAS1, can potentially prevent cancer progression (25, 26). In CRC, STAT3 expression has been observed in the crypt epithelial cell cytoplasm, and p-STAT3 is found in both the cytoplasm and nucleus (22). Furthermore, the inhibition of STAT3 expression made therapy-resistant CRC cells sensitive to 5-fluorouracil (27). p-STAT3 inhibition induces CRC cell apoptosis and cell cycle arrest (28). Our data suggested that TRIM6 potentially promoted CRC migration and invasion by modulating the status of STAT3 activation. This TRIM6-STAT3 regulatory network might function through the STAT3 phosphatase suppressor of cytokine signaling 2 (SOCS2); decreased levels of ubiquitous SOCS2 were detected in TRIM6 knockdown cells. Data from 70 samples from patients with CRC further confirmed that TRIM6 expression is positively correlated with STAT3 expression and negatively correlated with SOCS2.

## Materials And Methods

### Cell Culture

SW620 and HCT116 cells were bought from the Cell Bank of Shanghai Institute of Cell Biology, Chinese Academy of Sciences. The cells were maintained in DMEM (HyClone, Logan, UT, USA) with 10% fetal bovine serum (FBS) (Life Technology, Grand Island, NY, USA) at 37°C with 5% CO_2_.

### Manipulation of TRIM6 Expression by Lentivirus

Short hairpin RNA (shRNA) oligos targeting TRIM6 (shTRIM6-1: 5’-CCGGAGACAAGTGAGGTTT-3’; shTRIM6-2: 5’-GCTAAAGTATCTGGACCTT-3’; and shTRIM6-3: 5’-CCATGAATATAGGGCCTAT-3’) were cloned into the pLKO.1 (Addgene, Cambridge, MA, USA) vector between the AgeI and EcoRI sites. pLVX-puro (Clontech, Palo Alto, CA, USA) was used as the vector for a full-length TRIM6 construction. The corresponding lentivirus was produced in HEK-293T cells.

### Quantitative RT-PCR

Total RNA was extracted using the TRIzol reagent (Life Technology). cDNA was synthesized using a reverse transcription kit according to the instructions of the manufacturer (Fermentas, Hanover, MD, USA). SYBR Green Mix (Thermo Fisher Scientific, Rockford, IL, USA) was used for qRT-PCR with three replications per sample. The primers used were SOCS2 (5’-TGCCTTGCCTTCTTAGGTTC-3’ and 5’-CTTGGTTCCTTCCCACTTCTTC-3’) and GAPDH (5’-AATCCCATCACCATCTTC-3’and 5’-AGGCTGTTGTCATACTTC-3’). GAPDH was used to normalize relative mRNA levels.

### Western Blotting

RIPA buffer containing a protease inhibitor cocktail (Beyotime Biotech., Shanghai, China) was used to lyse cells. The cell was lysed for 30 min on ice. The extracted protein and Laemmli loading buffer were mixed at a 1:1 ratio before boiling for 5 min. Protein separation was then performed on 10% or 15% SDS-PAGE gel, and 5% skimmed milk was used for blocking for 1 h at room temperature. After the primary antibody (Appendix. Antibody list) incubation overnight, HRP-corresponding secondary antibodies (Beyotime Biotech.) were used. Signal exposure was conducted using the ECL kit (Pierce, Rockford, IL, USA).

### Wound-Healing Assay

The treated cells were seeded in a 6-well dish (8 × 10^5^ cells per well). Once cells achieved 85% confluence, a wound was scratched through the center of the dish. After washing with PBS, the cells were cultured in DMEM with 10% FBS. The images were taken at 0, 24, and 48 h post-wounding.

### Cell Invasion Assay

For the cell invasion assay, 8-μm-pore filters (Corning, New York, NY, USA) were used, and 1 mg/ml Matrigel (BD Biosciences) was used to precoat the upper chamber. The cells were grown to 50% confluence. After a 24-h serum-free medium incubation, the cells were suspended, and 5 × 10^4^ cells were seeded onto the upper chamber while the media with 10% FBS was filled in the lower chamber. At 24 h post-seeding, the non-migrating cells were washed away. The cells in the lower chamber were fixed in 4% PFA and stained with 0.5% crystal violet. The samples were imaged and counted with five random fields per chamber.

### Immunoprecipitation Assays

The cell lysates were incubated with anti-TRIM6 (Bioss, Bs-9165R), anti- SOCS2 (Abcam, Ab3692), or control IgG (Santa Cruz Biotech., Santa Cruz, CA, USA) for 1 h at 4°C. The cell mixture was then incubated with protein A/G-agarose (150 μg protein A) for 3 h at 4°C. The precipitates were then detected *via* Western blot analysis.

### *In Vivo* Metastasis Mouse Model

The experimental protocol was approved by the Animal Experimentation Ethics Committee of Zhabei Central Hospital of Jing'an District. We used 6-week-old male BALB/c nude mice housed under specific-pathogen-free conditions. To examine an experimental lung metastasis, HCT116 cells stably expressing TRIM6 shRNA (shTRIM6-1) or control shRNA (shNC) were introduced *via* lateral vein injection (1 × 10^6^ cells per mouse, n = 5 per group). Images of the mice were taken weekly using IVIS to monitor the metastatic tumor growth. Mice were sacrificed after 4 weeks, and lung metastases were counted. The lung tissue was fixed using 10% formalin and embedded in paraffin. Histological analysis was performed *via* hematoxylin and eosin (H&E) staining.

### Colorectal Cancer Tissue Samples and Immunohistochemical Staining

This study was approved by the Institutional Review Board of Zhabei Central Hospital of Jing'an District. Paraffin-embedded CRC specimens from patients treated at Zhabei Central Hospital of Jing'an District were enrolled in this study with their written consent. None of these patients had received radiotherapy or chemotherapy prior to surgery.

CRC specimen sections were deparaffinized and dehydrated using sequential diluted xylene and ethanol wash. The antigen was retrieved by treating slides in 0.01 M sodium citrate buffer (pH 6) for 15–20 min. The slides were then permeabilized in 3% hydrogen peroxide and blocked using 5% bovine serum albumin. Primary antibody anti-TRIM6 (ProteinTech, 11953-1-AP), anti-SOCS2 (Abcam, ab109245), or anti-p-STAT3 (Abcam, ab76315) was incubated overnight. The corresponding HRP secondary antibodies (Long Island Bio, Shanghai, China) were then used. The signal was developed using the DAB staining kit (Long Island Bio) along with hematoxylin staining. The intensity grading was calculated as follows: IRS = staining intensity (SI) × percentage of positive cells. The SI was ranked from 1 to 3 (negative to strong). Overexpression was defined as having a staining index >3. Staining was independently assessed by two investigators.

### Bioinformatics Analysis

Pathway and gene analyses were performed based on a CRC dataset (GSE14333) (29) using the GSEA version 2.0 from the Broad Institute at MIT. In our analysis, gene sets of fewer than 10 genes were excluded. The permutation test had a cutoff of 1,000 times. Significance was set at p < 0.01.

### Statistical Analysis

Statistical analysis was conducted using the GraphPad Prism software (version 6.0, San Diego, CA, USA). For the statistical evaluation of two groups or more, two-tailed Student’s t-test and one-way analysis of variance were used, respectively (*p < 0.05, **p < 0.01, ***p < 0.001).

## Results

### TRIM6 Knockdown Inhibits Colorectal Cancer Cell Migration and Invasion Both *In Vitro* and *In Vivo*

**Figure 1:**
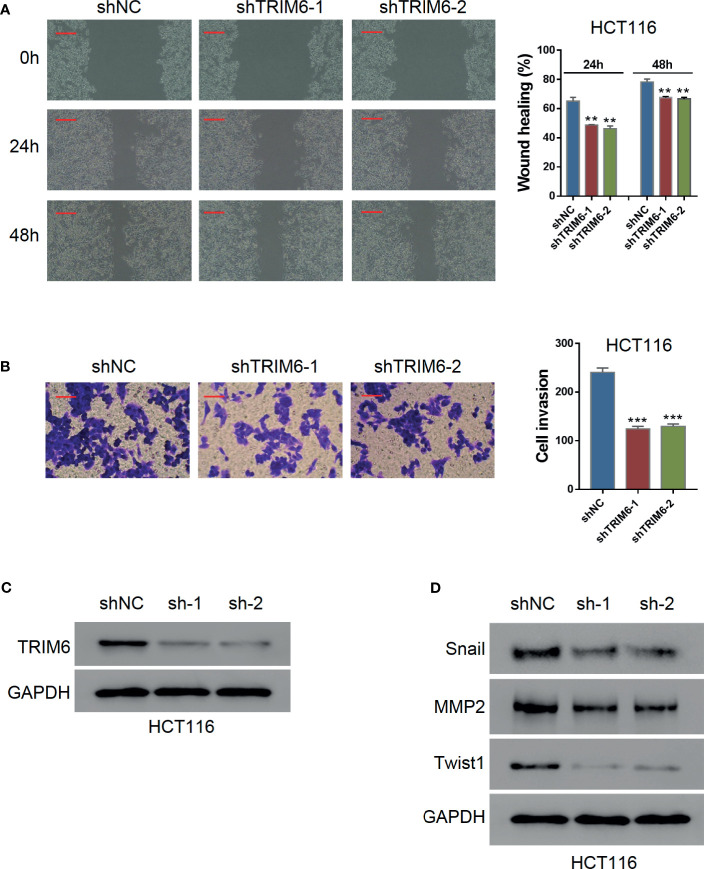
TRIM6 knockdown inhibits CRC cell migration and invasion in HCT116 cells. **(A)** Western blots images for validating TRIM6 knockdown by shRNAs. **(B)** Representative images and quantitative analysis of wound-healing assay at 0, 24, and 48 h post-scratching. shNC, negative control; shTRIM6-1, shRNA-1; shTRIM6-2, shRNA-2. Scale bar: 100 µm. **(C)** Representative images and quantitative analysis of the cell invasion assay. Scale bar: 100 µm. **(D)** Detecting the expression of Snail, MMP2, and Twist. The results are from at least three separate experiments. **P < 0.01, ***P < 0.001 *vs.* shNC.

To investigate the function of TRIM6 in CRC cell migration, we knocked down the TRIM6 expression in HCT116 cells using lentivirus-mediated shRNAs. Western blot validation showed that both shRNAs (shRNA-1 and shRNA-2) successfully downregulated the TRIM6 protein expression (**Figures 1A**; **S1A**). Wound-healing was significantly delayed in both TRIM6 knockdown groups (**Figure 1B**). Quantitatively, the healing gaps in the TRIM6 knockdown groups were around 20% larger than that of the shNC group (**Figure 1B**). Transwell invasion assay was then performed to identify the role of TRIM6 in cell invasion. Around half as much invaded cells were found in the TRIM6 knockdown groups compared with the control cells (**Figure 1C**). Moreover, we also detected the downregulation of the EMT markers, such as Snail and Twist1, and the invasion and migration marker MMP2 (**Figure 1D**). These results indicate that TRIM6 knockdown potentially inhibits CRC cell migration and invasion.

**Figure 2:**
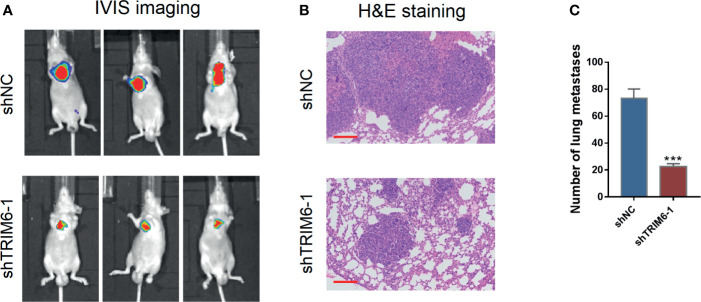
TRIM6 knockdown inhibits CRC lung metastasis in mice injected with HCT116 cells. **(A)** Representative bioluminescent images of mice (n = 5 per group) injected with the indicated cells taken at day 28. **(B)** Hematoxylin and eosin (H&E)-stained sections revealed that tumor cells metastasized to the alveoli of the lung in mice injected with the indicated cells. Scale bar: 200 µm. **(C)** Number of lung metastases. ***P < 0.001 *vs.* shNC.

We then explored the effect of TRIM6 in cell migration *in vivo* by monitoring the lung metastasis in mice injected with HCT116 stably expressing shRNA-1 and shNC. On IVIS imaging, shRNA-1-injected mice had lower lung metastatic signals (**Figure 2A**). Histologically, lung metastatic sites revealed cancerous features that were distinct from those of the surrounding lung tissues, such as nuclear and cytoplasmic enlargement, nuclear pleomorphism, and hyperchromatic and frequent multinucleation (**Figure 2B**). These metastatic sites were less frequently seen in the shRNA-1-injected group than in the shNC-injected group. The number of metastatic sites in the shRNA-1-injected group was around three times lower than those in the shNC-injected group (**Figure 2C**). These results indicate that TRIM6 dysfunction could potentially inhibit CRC metastasis.

### TRIM6 Overexpression Induces Colorectal Cancer Cell Migration *via* the Signal Transducer and Activator of Transcription 3 Pathway

**Figure 3:**
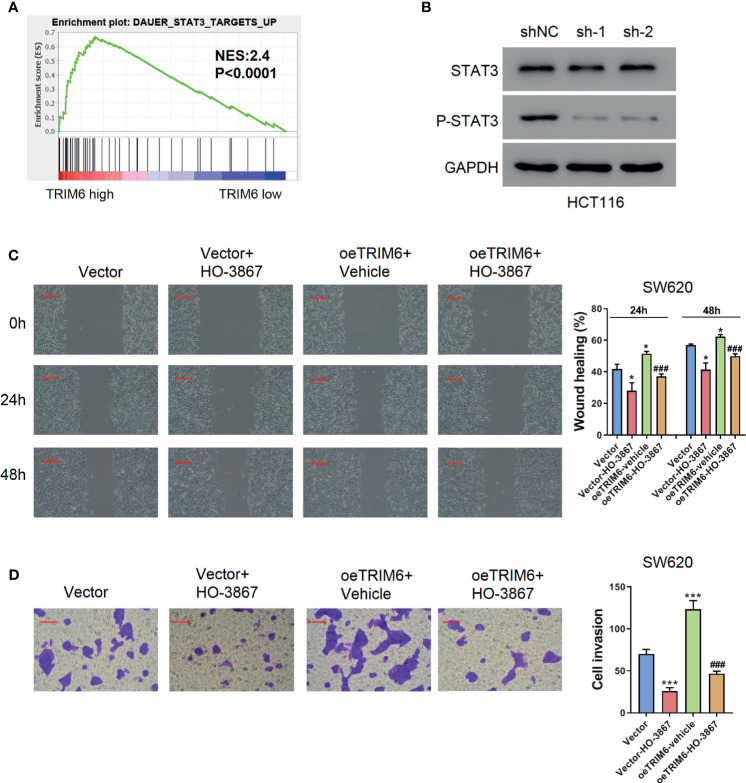
TRIM6 overexpression induces CRC cell migration *via* the STAT3 pathway in the SW620 cell line. **(A)** Enrichment plot of GSEA of the TRIM6 target. **(B)** STAT3 and p-STAT3 expression level detected *via* Western blot in TRIM6 knockdown HCT116 cells. **(C)** Representative images and quantitative analysis of the wound-healing assay in TRIM6-overexpressed SW620 cells with/without HO-3867 treatment at 0, 24, and 48 h post-scratching. Scale bar: 100 µm. **(D)** Representative images and quantitative analysis of the cell invasion assay in TRIM6-overexpressed SW620 cells with/without HO-3867 treatment. Scale bar: 100 µm. *P < 0.05, ***P < 0.001 *vs.* Vector; ^###^P < 0.001 *vs.* oeTRIM6 + Vehicle. The results are from at least three separate experiments.

TRIM6 knockdown showed an inhibitory effect on CRC cell migration. To further test how TRIM6 promotes cell migration, we conducted a GSEA analysis on TRIM6. Interestingly, TRIM6 expression was positively correlated with several cancer-related pathways (**Table S1**), including NF-κB, CTNNB1, and STAT3 signaling (**Figure 3A**). Our preliminary data showed that TRIM6 knockdown had little effects on the phosphorylation of NF-κB p65 and the nuclear translocation of β-catenin (data not shown). Furthermore, although STAT3 expression was not affected in TRIM6 knockdown HCT116 cells, the phosphorylated STAT3 level was significantly lower than that in the control cells (**Figure 3B**). Thus, we supposed that TRIM6 may promote cell migration *via* the STAT3 signaling pathway. The genes included in DAUER_STAT3_TARGETS_UP affected by TRIM6 expression levels are listed in **Table S2**. To test this hypothesis, SW620 cells were overexpressed with TRIM6 (**Figure S1B**) and treated with STAT3 phosphorylation inhibitor HO-3867. TRIM6 overexpression in SW620 was able to induce cell migration in both the wound-healing and cell invasion assays (**Figures 3C, D**). After inhibiting STAT3 phosphorylation, the SW620 cells had a delayed cell migration (**Figures 3C, D**). Interestingly, HO-3867 rescued the TRIM6 overexpression-induced CRC cell migration phenotype.

### TRIM6 May Promote Signal Transducer and Signal Transducer and Activator of Transcription 3 Phosphorylation *via* Suppressor of Cytokine Signaling 2

**Figure 4:**
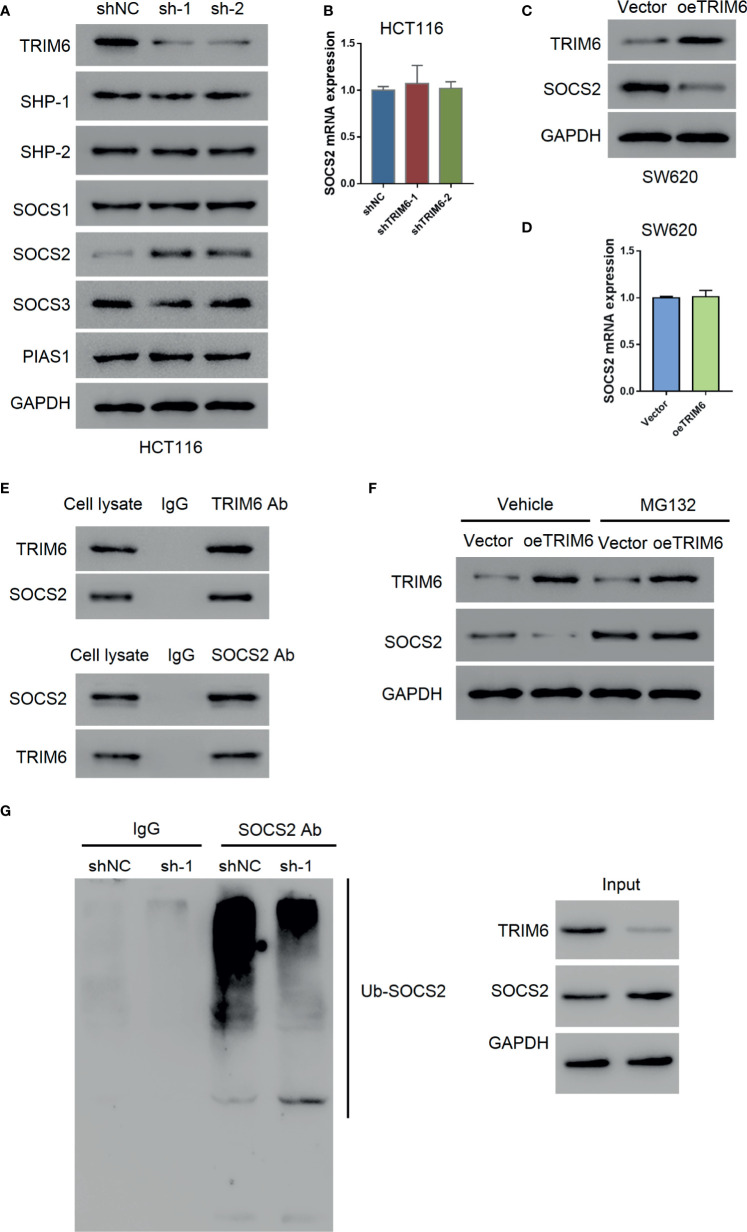
TRIM6 may promote STAT3 phosphorylation *via* SOCS2. **(A)** Protein expression of STAT3 phosphatases (SHP-1, SHP-2), SOCS1, SOCS2, SOCS3, and PIAS1 in TRIM6 knockdown HCT116 cells. **(B)** SOCS2 mRNA expression level measured *via* qRT-PCR in TRIM6 knockdown HCT116 cells. SOCS2 protein **(C)** and mRNA expression levels **(D)** in TRIM6-overexpressed SW620 cells detected *via* Western blot and qRT-PCR, respectively. **(E)** CO-IP analysis between TRIM6 and SOCS2. **(F)** TRIM6 and SOCS2 expression levels in TRIM6-overexpressed SW620 cells with/without MG132 treatment. **(G)** IP analysis of SOCS2 ubiquitination in TRIM6 knockdown HCT116 cells. The results are from at least three separate experiments.

Our previous results indicated that TRIM6 promotes CRC cell migration *via* STAT3 signaling. To further investigate the regulatory mechanism of TRIM6 on STAT3, we examined the expression level of STAT3-negative regulators (SHP-1, SHP-2, SOCS1, SOCS2, SOCS3, and PIAS1). Among these, only the SOCS2 protein expression was decreased in TRIM6 knockdown HCT116 cells (**Figure 4A**). Thus, we focused on SOCS2. The mRNA expression level of SOCS2 remained unchanged (**Figure 4B**), whereas the protein expression of SOCS2 was upregulated in TRIM6-overexpressed SW620 cells without altering its mRNA expression level (**Figures 4C, D**). Meanwhile, the expression level of other STAT3-negative regulators remained unchanged.

SOCS2 plays a key role in inhibiting JAK-STAT signaling by degrading the signaling molecules *via* ubiquitination (30). CO-IP analysis with HCT116 cell lysate revealed a potential interaction between TRIM6 and SOCS2 (**Figure 4E**). To further understand the relationship between TRIM6 and SOCS2, we used the proteasome inhibitor MG132 to treat the TRIM6-overexpressed SW620 cells. We observed that MG132 treatment blocked the TRIM6 overexpression-induced reduction of SOCS2 protein expression in SW620 cells (**Figure 4F**). In TRIM6 knockdown HCT116 cells, because of the increase in the SOCS2 protein expression, SOCS2 ubiquitination was largely inhibited (**Figure 4G**). These results indicate that TRIM6 can negatively promote SOCS2 expression through ubiquitination.

### TRIM6 Expression Positively Correlates With p-Signal Transducer and Activator of Transcription 3 and Negatively Correlates With SOCS2 in Samples From Patients With Colorectal Cancer

**Figure 5:**
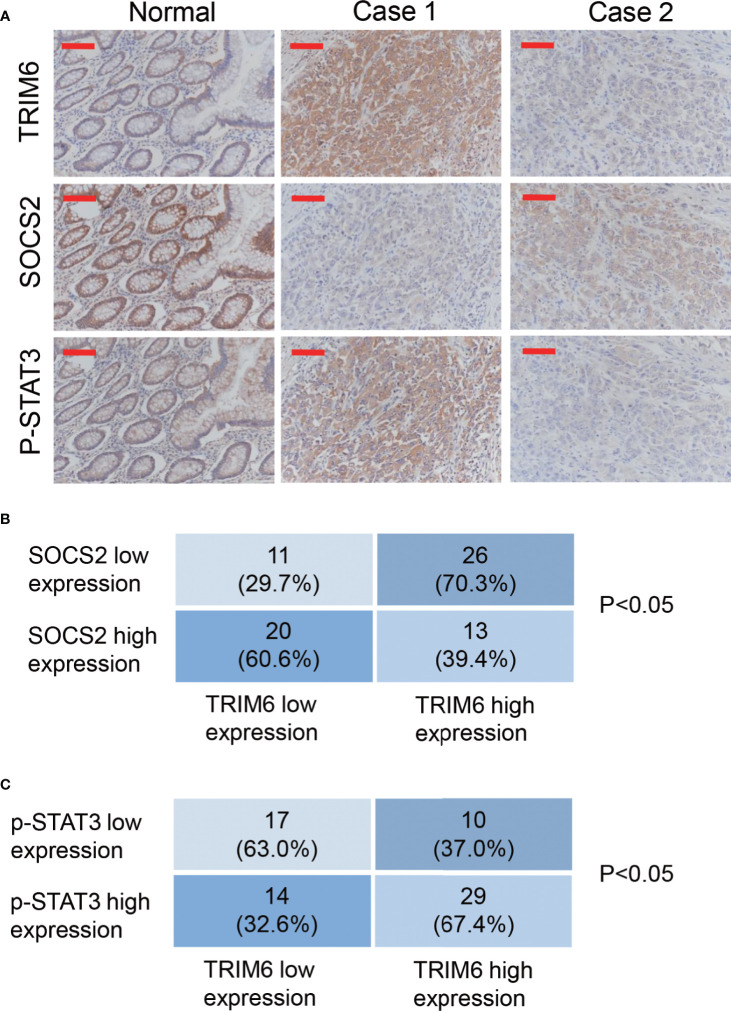
TRIM6 expression positively correlated with p-STAT3 and negatively correlated with SOCS2 in the CRC samples. **(A)** Representative immunohistochemical staining of TRIM6, SOCS2, and p-STAT3 in paraffin-embedded normal and CRC specimens (Case 1 and Case 2). n = 70. Scale bar: 100 µm. **(B, C)** Statistical analysis of CRC tissues under different staining conditions.

To further investigate the function of TRIM6 in patients with CRC, we collected 70 paraffin-embedded CRC specimens from patients treated at Zhabei Central Hospital of Jing'an District between 2012 and 2014. Patient information is shown in **Table S3**. Immunohistochemical staining and statistical analysis further confirmed our previous results. In the majority of the CRC specimens collected, TRIM6 expression was positively correlated with a phosphorylated STAT3 expression, whereas TRIM6 expression was negatively correlated with SOCS2 expression (**Figure 5**).

## Discussion

TRIM6 protein has been reported to promote cell cycle progression and CRC cell proliferation (16). In the *in vitro* and *in vivo* functional assays, we observed that TRIM6 promotes CRC migration. Our findings, combined with that of a previous study (16), support the important roles of TRIM6 in CRC growth and metastasis.

In the current study, we used two independent CRC cell lines, HCT116 and SW620, which have previously established their intravasation capabilities and suitability for building a CRC metastasis model (31). Studies have also shown that the HCT116 cell lines have a mutation in TGFβRII, which inactivates kinase activity (32). However, the TRIM family has also been shown to affect cancer progression *via* TGF-β signaling (33). Moreover, a previous study comparing 10 CRC cell lines found that SW620 has the lowest TRIM6 expression while being at a physiologically relevant level (34). Therefore, to investigate the function of TRIM6 in CRC metastasis under restored TGF-β signaling, we chose to use SW620 cells for TRIM6 overexpression studies. *In vitro*, TRIM6 knockdown in HCT116 cells inhibited cell migration and invasion (**Figure 1**). Furthermore, the difference caused by TRIM6 knockdown was more noticeable at 24 h post wound creation, suggesting the involvement of other migration related factors at later times post the scratch formation. Meanwhile, whether secondary pathways or TRIM6 directly results in the downstream effects needs to be further verified. Epithelial-mesenchymal transition (EMT) is strongly implicated in tumor cell migration, invasion, and metastasis (35). Snail1 and Twist1, two markers of EMT, and MMP2, a marker of invasion and migration, which could degrade the extracellular matrix, were downregulated in TRIM6 knockdown CRC cells, which is consistent with a study on TRIM6 in renal fibrosis (36). In this perspective, TRIM6 stimulates the EMT and production of MMP2, which could facilitate tumor cell migration, invasion, and metastasis, thus, causes tumor recurrence. *In vivo*, we observed fewer CRC lung metastatic sites in the TRIM6 knockdown HCT116 cell-injected mouse CRC model (**Figure 2**). In contrast, TRIM6-overexpressed SW620 cells induced cell migration and invasion (**Figure 3**). Both *in vitro* and *in vivo* experiments revealed that TRIM6 promotes CRC migration. Evidence suggested that cell hyperproliferation is another key event contributing to the anticancer response (16). The possible effect of TRIM6 on cell proliferation could not be ruled out and further work will be necessary.

Regarding downstream signaling, TRIM6 expression was positively correlated with STAT3 signaling on GSEA (**Table S1**). Evidence has supported the critical role of STAT3 singling in the migration and invasion of cancer cells (24). In line with this, we found that the STAT3 phosphorylation inhibitor can rescue the TRIM6 overexpression phenotype, showing a delayed migration and decreased cell invasion (**Figure 3**). We further showed that TRIM6 expression level was correlated with the protein expression level of SOCS2 (a negative regulator of STAT3) whose expression was reduced in CRC tissues (37). TRIM family proteins are reported to have a E3 ubiquitin ligase activity (38). Recent studies have reported that TRIM6 promoted the ubiquitination of TIS21 (16) and TSC1-TSC2 (36). TRIM6 interacts with SOCS2, and the level of SOCS2 ubiquitination was lower in TRIM6 knockdown HCT116 cells (**Figure 4**), whereas the ubiquitination site on SOCS2 is to be identified. Furthermore, previous studies in dendritic cells have shown that SOCS2 silencing caused a hyperphosphorylation of STAT3 (39). STAT3 hyperactivation was also reported to correlate with SOCS2 silencing in ovarian and breast cancers (25). Based on these findings, we propose the possibility of a TRIM6-STAT3-SOCS2 regulatory signaling network involved in CRC metastasis. TRIM6 dysfunction causes an increased SOCS2 expression, resulting in a decreased level of STAT3 activation, CRC cell migration, and invasion. On the other hand, TRIM6 overexpression causes SOCS2 expression to be downregulated. This results in an increased cell migration and invasion, as well as CRC metastasis. Inhibition of the STAT3 signaling could decrease the expression of Snail1, Twist1, and MMP2 (40, 41). Here, TRIM6 knockdown decreased the expression of Snail1, Twist1, and MMP2 (**Figure 1**), which may be due to the decreased phosphorylation of STAT3 through inhibiting the ubiquitination of SOCS2.

Our findings were also supported by the samples from patients with CRC. From the 70 collected samples, Fisher’s exact test showed that the TRIM6 levels were correlated with the tumor size, clinical stage, and metastasis (**Table S4**). In addition, we found that TRIM6 expression was positively correlated with STAT3 phosphorylation and negatively correlated with SOCS2 expression (**Figure 5**). According to the analysis of GSE14333 (**Figure S2A**) and a recent report (16), TRIM6 expression was negatively correlated with the overall survival of CRC. By using GEPIA (42), we found that TRIM6 expression was not correlated with the overall survival of CRC (**Figure S2B**), but it was negatively correlated with the disease-free survival of CRC (**Figure S2C**), suggesting that TRIM6 could constitute a useful prognostic marker to identify patients who are more likely to have disease recurrence and are, thus, good candidates to receive an aggressive adjuvant treatment. However, future prospective studies are needed to determine its accuracy and efficiency on predicting the recurrence of patients with CRC in order to tailor adjuvant treatment. The restricted patient sample size limited our findings. Further retrospective analysis with a larger sample size can be carried out to investigate the role of TRIM6 in the overall survival of patients. Furthermore, the cell line with a lower TRIM6 expression (SW620) was metastatic, indicating the complicated regulation of cancer cell metastasis. Our study suggested that TRIM6 may serve as a CRC therapeutic target for those patients with a high expression of TRIM6 in the future. For those with a low expression of TRIM6, other therapeutic targets, such as Wnt/β-catenin and NF-κB (43, 44), should be considered.

In conclusion, our study was the first to reveal that TRIM6 promotes CRC cell migration and invasion *via* the SOCS2-STAT3 signaling. Our results uncover the potential of TRIM6 as a potential CRC therapeutic target.

## Data Availability Statement

The original contributions presented in the study are included in the article/**Supplementary Material**. Further inquiries can be directed to the corresponding authors.

## Ethics Statement

The studies involving human participants were reviewed and approved by the Institutional Review Board of Zhabei Central Hospital of Jing'an District. The patients/participants provided their written informed consent to participate in this study. The animal study was reviewed and approved by the Animal Experimentation Ethics Committee in Zhabei Central Hospital of Jing'an District.

## Author Contributions

MZ, XC, and HZ designed the experiments. HZ, JH, and MC performed the experiments. HZ and BL performed the statistical analysis. HZ and JH wrote the manuscript. All authors contributed to the article and approved the submitted version.

## Funding

This study was supported by 2019 Research Projects of Shanghai Municipal Health Commission (201940198).

## Conflict of Interest

The authors declare that the research was conducted in the absence of any commercial or financial relationships that could be construed as a potential conflict of interest.

## Publisher’s Note

All claims expressed in this article are solely those of the authors and do not necessarily represent those of their affiliated organizations, or those of the publisher, the editors and the reviewers. Any product that may be evaluated in this article, or claim that may be made by its manufacturer, is not guaranteed or endorsed by the publisher.
