# Supplementary material for: Tripartite Motif Protein 6 Promotes Colorectal Cancer Cell Migration and Metastasis via SOCS2-STAT3 Signaling
Source: Front Oncol. 2021 Sep 13;11:695525. doi: 10.3389/fonc.2021.695525 (PMC8473888; doi:10.3389/fonc.2021.695525)
Supplement: Supplementary file 1 [file DataSheet_1.docx]

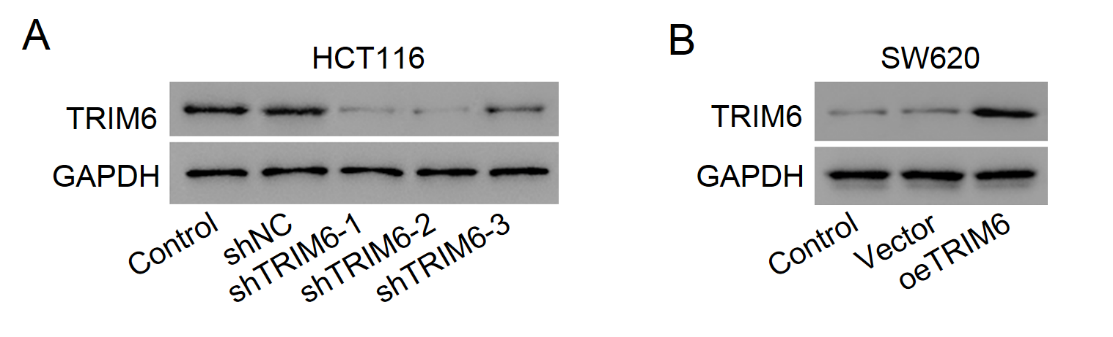


**Figure S1.** Western blot detection of TRIM6 expression level in TRIM6 knockdown HCT116 cells and TRIM6 overexpressed SW620 cells.


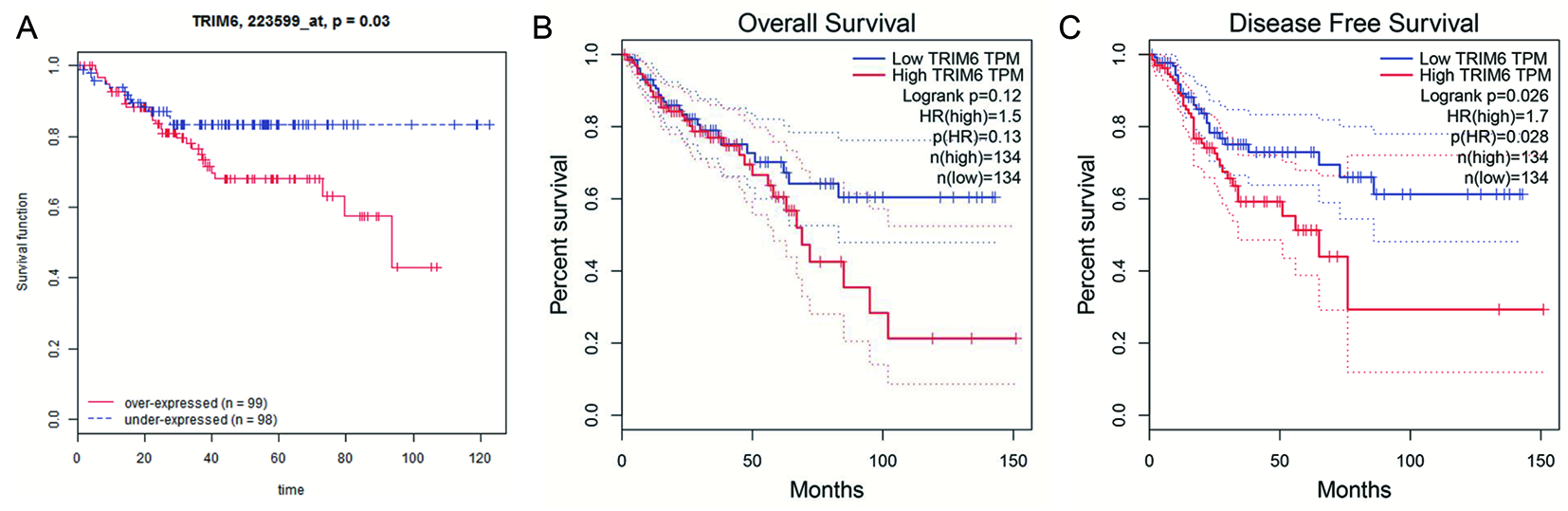


**Figure S2.** Survival outcome of patients with CRC. (A) Overall survival of patients with CRC in GSE14333 dataset. (B) Overall and (C) Disease free survival of patients with CRC in Gene Expression Profiling Interactive Analysis (GEPIA) database.

**Table S1.** GSEA analysis.

| NAME | SIZE | ES | NES | NOM p-val | FDR q-val | FWER p-val | RANK AT MAX |
| --- | --- | --- | --- | --- | --- | --- | --- |
| HALLMARK_TNFA_SIGNALING_VIA_NFKB | 193 | 0.587621 | 2.57544 | 0 | 0 | 0 | 4581 |
| ONDER_CDH1_SIGNALING_VIA_CTNNB1 | 79 | 0.6550719 | 2.5589125 | 0 | 0 | 0 | 3211 |
| AZARE_NEOPLASTIC_TRANSFORMATION_BY_STAT3_UP | 113 | 0.6197811 | 2.5573137 | 0 | 0 | 0 | 3032 |
| PHONG_TNF_RESPONSE_VIA_P38_PARTIAL | 151 | 0.5821437 | 2.4832382 | 0 | 0 | 0 | 3695 |
| SCHOEN_NFKB_SIGNALING | 30 | 0.7500306 | 2.4085038 | 0 | 0 | 0 | 3539 |
| DAUER_STAT3_TARGETS_UP | 45 | 0.6677081 | 2.3995726 | 0 | 0 | 0 | 3242 |
| WIERENGA_STAT5A_TARGETS_DN | 185 | 0.5477578 | 2.3884192 | 0 | 0 | 0 | 3095 |
| DAUER_STAT3_TARGETS_DN | 47 | 0.6657052 | 2.3656862 | 0 | 0 | 0 | 3695 |
| HINATA_NFKB_TARGETS_KERATINOCYTE_UP | 87 | 0.5912039 | 2.359434 | 0 | 0 | 0 | 4643 |
| HAN_JNK_SINGALING_DN | 36 | 0.6814525 | 2.3218877 | 0 | 0 | 0 | 3138 |

**Table S2.** DAUER_STAT3_TARGETS_UP affected by TRIM6 expression levels.

|  | Gene | Rank in gene list | Rank metric score | Running ES | Correlation Score |
| --- | --- | --- | --- | --- | --- |
| 1 | AKAP12 | 74 | 0.51 | 0.0529 | 0.5096554 |
| 2 | SAMD4A | 126 | 0.488 | 0.1046 | 0.48839664 |
| 3 | HEG1 | 438 | 0.412 | 0.1348 | 0.41187295 |
| 4 | BCL6 | 642 | 0.378 | 0.1666 | 0.37753794 |
| 5 | KLF11 | 651 | 0.376 | 0.208 | 0.37602156 |
| 6 | ABCA1 | 678 | 0.372 | 0.248 | 0.37218165 |
| 7 | CCL2 | 736 | 0.363 | 0.2854 | 0.3628537 |
| 8 | SLC2A3 | 760 | 0.36 | 0.3242 | 0.3598375 |
| 9 | GFPT2 | 828 | 0.351 | 0.3598 | 0.35079357 |
| 10 | THBS1 | 1068 | 0.322 | 0.3837 | 0.32200772 |
| 11 | STAT2 | 1107 | 0.317 | 0.417 | 0.31746426 |
| 12 | DUSP5 | 1257 | 0.3 | 0.4429 | 0.30029154 |
| 13 | SOCS3 | 1482 | 0.275 | 0.4624 | 0.2754807 |
| 14 | CEBPD | 1639 | 0.26 | 0.514 | 0.25997078 |
| 15 | CTSB | 1748 | 0.251 | 0.5366 | 0.25145635 |
| 16 | MAFF | 1814 | 0.246 | 0.5606 | 0.24562572 |
| 17 | PFKFB3 | 2251 | 0.209 | 0.5621 | 0.20885146 |
| 18 | NPC1 | 2392 | 0.199 | 0.5772 | 0.19854471 |
| 19 | ZFP36 | 2424 | 0.196 | 0.5974 | 0.19621493 |
| 20 | PLIN3 | 2582 | 0.186 | 0.6103 | 0.18639103 |
| 21 | ICAM1 | 2800 | 0.174 | 0.6189 | 0.17419365 |
| 22 | ATF3 | 2802 | 0.174 | 0.6381 | 0.1741898 |
| 23 | ADM | 3059 | 0.159 | 0.643 | 0.15871711 |
| 24 | IGFBP1 | 3120 | 0.155 | 0.6573 | 0.15540259 |
| 25 | UGCG | 3242 | 0.148 | 0.6677 | 0.14787859 |

ES: enrichment score

**Table S3.** Patient information.

| Characteristic | Cases | % |
| --- | --- | --- |
| Gender |  |  |
| Male | 38 | 54.3 |
| Female | 32 | 45.7 |
| Age (years) |  |  |
| ≥65 | 25 | 35.7 |
| <65 | 45 | 64.3 |
| Tumor size (cm) |  |  |
| ≥4.0 | 48 | 68.6 |
| <4.0 | 22 | 31.4 |
| Pathologic differentiation |  |  |
| Well/Moderate | 50 | 71.4 |
| Poor | 20 | 28.6 |
| Clinical stage |  |  |
| I/II | 29 | 41.4 |
| III | 41 | 58.6 |
| Metastasis |  |  |
| yes | 27 | 38.6 |
| no | 43 | 61.4 |
| TRIM6 expression |  |  |
| Low | 31 | 44.3 |
| High | 39 | 55.7 |

**Table S4.** Correlation of TRIM6 expression with different clinicopathological features in colorectal cancer tissues (n=70).

| Characteristic | TRIM6 | | *P*-value |
| --- | --- | --- | --- |
|  | Low (n=31) | High (n=39) |  |
| Gender |  |  | 0.6338 |
| Male | 18 | 20 |  |
| Female | 13 | 19 |  |
| Age (years) |  |  | 0.8022 |
| ≥65 | 12 | 13 |  |
| <65 | 19 | 26 |  |
| Tumor size (cm) |  |  | 0.0094** |
| ≥4.0 | 16 | 32 |  |
| <4.0 | 15 | 7 |  |
| Pathologic differentiation |  |  | 0.1837 |
| Well/Moderate | 25 | 25 |  |
| Poor | 6 | 14 |  |
| Clinical stage |  |  | 0.0154* |
| I/II | 18 | 11 |  |
| III | 13 | 28 |  |
| Metastasis |  |  | 0.0252* |
| no | 24 | 19 |  |
| yes | 7 | 20 |  |

Clinicopathological features were assessed using the Fisher’s exact test. **P*<0.05, ***P*<0.01.

**Appendix.**Antibody list

| **Primary antibody** | **Company** | **Catalog No.** |
| --- | --- | --- |
| TRIM6 | Proteintech | 11953-1-AP |
| STAT3 | Abcam | Ab19352 |
| p-STAT3 | Abcam | Ab76315 |
| Snail | Abcam | Ab59208 |
| MMP2 | Abcam | Ab97779 |
| Twist1 | Abcam | Ab175430 |
| SHP-1 | Abcam | Ab32559 |
| SHP-2 | Abcam | Ab32083 |
| SOCS1 | Abcam | Ab62584 |
| SOCS2 | Abcam | Ab109245 |
| SOCS3 | Abcam | Ab16030 |
| PIAS1 | Abcam | Ab32219 |
| Ubiquitin | Abcam | Ab7780 |
| GAPDH | Cell Signaling Technology | #5174 |
